# N-Acetylaspartate and Choline Metabolites in Cortical and Subcortical Regions in Clinical High Risk Relative to Healthy Control Subjects: An Exploratory 7T MRSI Study

**DOI:** 10.3390/ijms24097682

**Published:** 2023-04-22

**Authors:** Ahmad Mayeli, Sabine A. Janssen, Chloe A. Huston, Julia S. Rupp, Kamakashi Sharma, Chan-Hong Moon, Ahmadreza Keihani, Hoby P. Hetherington, Fabio Ferrarelli

**Affiliations:** 1 Department of Psychiatry, University of Pittsburgh, Pittsburgh, PA 15213, USA; 2 Department of Radiology, University of Pittsburgh, Pittsburgh, PA 15213, USA

**Keywords:** NAA, choline, clinical high risk for psychosis, DLPFC, brain cortical regions

## Abstract

N-acetylaspartate (NAA) and choline (Cho) are two brain metabolites implicated in several key neuronal functions. Abnormalities in these metabolites have been reported in both early course and chronic patients with schizophrenia (SCZ). It is, however, unclear whether NAA and Cho’s alterations occur even before the onset of the disorder. Clinical high risk (CHR) individuals are a population uniquely enriched for psychosis and SCZ. In this exploratory study, we utilized 7-Tesla magnetic resonance spectroscopic imaging (MRSI) to examine differences in total NAA (tNAA; NAA + N-acetylaspartylglutamate [NAAG]) and major choline-containing compounds, including glycerophosphorylcholine and phosphorylcholine [tCho], over the creatine (Cre) levels between 26 CHR and 32 healthy control (HC) subjects in the subcortical and cortical regions. While no tCho/Cre differences were found between groups in any of the regions of interest (ROIs), we found that CHR had significantly reduced tNAA/Cre in the right dorsal lateral prefrontal cortex (DLPFC) compared to HC, and that the right DLPFC tNAA/Cre reduction in CHR was negatively associated with their positive symptoms scores. No tNAA/Cre differences were found between CHR and HC in other ROIs. In conclusion, reduced tNAA/Cre in CHR vs. HC may represent a putative molecular biomarker for risk of psychosis and SCZ that is associated with symptom severity.

## 1. Introduction

Schizophrenia (SCZ) is a psychiatric disorder that affects approximately 1% of the population, and is a major cause of disability worldwide [1]. Identifying neurobiological and clinical risk factors for SCZ is, therefore, a high priority for research and public health [2]. Clinical high risk for psychosis (CHR) is a population uniquely enriched for risk of psychosis and SCZ. The CHR state includes individuals who are experiencing social dysfunction, cognitive impairments, and subsyndromal psychotic symptoms [3]. These individuals have a high risk of developing a psychiatric disorder, given that approximately 15–30% will transition to SCZ [4]. Thus, investigating CHR can lead to the discovery of early pathophysiological and risk/susceptibility biomarkers of SCZ. These findings can, in turn, contribute to establishing possible preventative measures and developing treatment options to help alleviate distressing symptoms.

Currently, identifying individuals at CHR, as well as diagnosis of SCZ, is entirely based on the subjective interpretation of clinical symptoms, as objective biological tests are not available [5]. As such, the interest in examining brain metabolites to help discover biomarkers of CHR and SCZ has grown in recent years [5]. One of the metabolites that has been increasingly investigated is choline (Cho). Cho plays a crucial role in generating membrane phospholipids that are essential in the human brain [6]; an elevated Cho signal is thought to point to an increase in membrane synthesis and turnover [7]. In SCZ, significantly higher levels of Cho have been observed in the dorsolateral prefrontal cortex (DLPFC) of chronic SCZ and first-episode psychosis (FEP) patients compared to healthy control (HC) subjects [8,9]. SCZ patients have also been found to have elevated Cho levels in the left caudate nucleus [10] and insular cortex [11] relative to controls. Concerning high risk populations, some studies have reported no differences in Cho levels between CHR and HC individuals in the thalamus [12,13], medial temporal region [14], the anterior cingulate cortex (ACC) [15,16,17], and the dorsolateral prefrontal cortex (DLPFC) [12,13,18]. However, a limitation of these studies was that some brain regions known to be implicated in SCZ have been less examined (e.g., insula, caudate); further, none of these studies employed ultrahigh field (≥7 Tesla, 7T) spectroscopy.

In addition to Cho, the amino acid derivative N-acetylaspartic acid (NAA) is another prominent metabolite in the brain [19]. NAA is known to be involved in the energy metabolism of neurons within the brain [20], and in glutamate receptor activity [21]. A decrease in NAA levels has been reported in SCZ [22]. Specifically, in chronic SCZ, NAA levels were significantly lower than controls in the frontal lobe, the temporal lobe, the thalamus, and the parietal lobe, while in FEP, NAA was lower than controls in the frontal lobe, the anterior cingulate cortex, the thalamus, and the DLPFC [22,23,24]. Few MRS studies have investigated NAA abnormalities in individuals at risk for psychosis; they found mostly negative results in the hippocampus and the anterior cingulate [14], and some significant or trend-level NAA reductions in the thalamus [12,25,26]. Findings in the prefrontal cortex have been mixed [13,26,27,28], although none of these studies employed 7T spectroscopy.

The use of magnetic resonance spectroscopic imaging (MRSI) allows the acquisition of data from multiple voxels simultaneously [29,30,31]. Compared to single-voxel MRS, which is limited to acquiring data from a single brain region at a time, spectral data from MRSI can be from a single slice, multiple slices, or multiple volumes simultaneously [31]. Thus, MRSI is more efficient than single-voxel MRS [32], and provides greater spatial information regarding metabolites of interest [33]. Additionally, MRSI allows for the direct comparison of neurotransmitter or metabolite levels because the time of acquisition is the same for the selected brain regions [32]. Relatedly, using ultrahigh field (≥7T) spectroscopy can offer a more precise quantification of the assessed metabolites [34]. Recent studies compared the measurement of total N-acetylaspartate (tNAA) using 7T and 3T MRS, and found a lower Cramer–Rao lower bound (CRLB)—a useful metric to evaluate the quality of the metabolite assessment, with lower values reflecting better measurements—for tNAA at 7T compared to 3T [35,36]. In this exploratory study, we employed 7T MRSI to measure tNAA and tCho in both cortical and subcortical brain regions in CHR and HC groups. Our overarching goal was to characterize abnormalities in these parameters in individuals at risk for SCZ, and to examine the association between those neurometabolite abnormalities and clinical symptoms in CHR vs. HC subjects.

## 2. Results

### 2.1. Clinical and Demographic Measures

Demographic variables and clinical measurements for HC and CHR groups are provided in Table 1. Twenty-six CHR individuals (fifteen females, age: 19.93 ± 3.16) and thirty-two HC subjects (eighteen females, age: 21.48 ± 4.98) were included in the study. By employing an unpaired *t*-test, we found no significant age differences across the groups (t(56) = −1.38, *p* = 0.172). Additionally, a chi-squared test revealed no sex differences between HC and CHR (X^2^ (1, N = 58) = 0.012, *p* = 0.912). The average scale of prodromal symptoms (SOPS) scores for the CHR group are reported in Table 1.

### 2.2. Group Differences in tNAA/Cre and tCho/Cre

**Figure 1:**
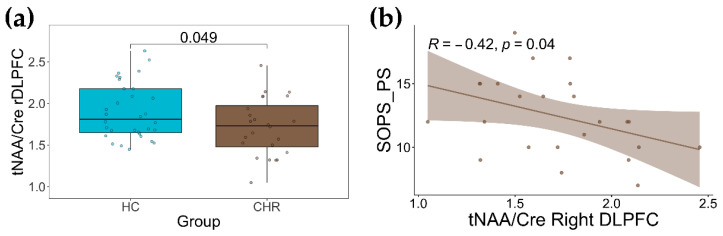
(**a**) group difference in [N-acetylaspartate (NAA) + N-acetylaspartylglutamate (NAAG)/creatine] (tNAA/Cre) levels in the right dorsolateral prefrontal cortex (DLPFC) between clinical high risk for psychosis (CHR) and healthy control (HC). (**b**) Negative correlation between scale of prodromal symptoms (SOPS) positive symptoms and tNAA/Cre levels in the right DLPFC.

We performed a one-way analysis of covariances (ANCOVAs) for tCho/Cre and tNAA/Cre between groups, and corrected them for individual age, sex, grey matter, and white matter fractions (Table 2). We found no significant differences in tCho/Cre levels in any brain regions of interest (ROIs) between CHR and HC (Table 2). In contrast, we found a lower tNAA/Cre, which was significant (*p* = 0.049; Cohen’s effect size = −0.546), in CHR relative to HC in the right DLPFC (Table 2 and Figure 1a). Further, the post hoc power analyses indicated the right DLPFC tNAA/Cre difference between groups (N = 58 total sample size) had a power level of >99%. There were no other differences in tNAA/Cre levels between CHR and HC in any other ROIs.

### 2.3. Association with Clinical Measurements

By performing Spearman correlations between tNAA/Cre levels in CHR and SOPS subscales, we found a significant correlation between tNAA/Cre levels and SOPS positive symptoms (rho = −0.42 and *p* = 0.040; Figure 1b). There was no correlation between right DLPFC tNAA/Cre levels and any other SOPS subscales (i.e., negative symptoms rho = 0.23 and *p* = 0.299; disorganized symptoms, rho = 0.20 and *p* = 0.371; general symptoms, rho = 0.15 and *p* = 0.490).

## 3. Discussion

By employing 7T MRSI, we computed tNAA/Cre and tCho/Cre levels in several cortical and subcortical brain ROIs in CHR and HC individuals. The ROIs selected for this study included both brain regions that previous work has shown to be altered in individuals at CHR or patients with SCZ, and brain regions which have been previously understudied in these patients. Our analysis revealed that tCho/Cre levels were not significantly different between the two study groups in any ROIs. In contrast, CHR individuals had lower tNAA/Cre in the right DLPFC compared to HC subjects. Furthermore, reduced tNAA/Cre levels in the right DLPFC in CHR were negatively associated with the severity of their positive, psychotic symptoms.

A first important finding of this study was that tCho/Cre levels did not differ between CHR and HC groups in any of the ROIs investigated. Our finding aligns with much of the existing literature that reported no Cho differences between CHR and HC groups in the PFC [13,15,37,38], thalamus [12,13,39], ACC [13,15,16,39], and DLPFC [12,13,18]. In addition, our study provided neurometabolite measurements for brain regions, which, to the best of our knowledge, have either not been previously investigated (insula), or have been understudied (caudate) in individuals at CHR. Given that the insula and the caudate are two brain regions known to be implicated in the pathophysiology of SCZ, and that Cho alterations have been previously reported in those regions [10,40], these negative results further corroborate the notion that alterations in Cho levels are not present in at-risk individuals, and are unlikely to be detectable before the diagnosis of SCZ.

We reported no significant decreases in NAA in the thalamus, the caudate, and the ACC in CHR vs. HC. These findings are consistent with negative findings by one MRS study examining NAA in the ACC of FES and ultrahigh risk (UHR) vs. HC [15], as well as two other spectroscopy recent studies in FEP [41] and chronic SCZ patients [42]. In contrast, two MRS meta-analyses found significant or trend-level NAA reductions in the thalamus of CHR vs. HC groups [12,25]. Several factors, including the heterogeneity of the CHR population, differences in sample sizes, and strength of the scanner (3T vs. 7T), may have contributed to these discrepant findings. In this regard, recent work comparing the measurement of total N-acetylaspartate (tNAA) using 7T and 3T MRS found a lower CRLB, which is thought to reflect a more precise measurement, for 7T vs. 3T [36]. Nonetheless, additional MRS studies are needed to further explore these inconsistent NAA findings in the thalamus. Relatedly, another meta-analysis study has shown reduced NAA levels in the ACC, thalamus, and frontal lobe in FEP, and in the frontal lobe, parietal lobe, temporal lobe, and thalamus in chronic schizophrenia, and showed progressive reduction in NAA in schizophrenia [23], which may indicate worsening neuronal integrity and function throughout the course of the disorder [43]. Together, these findings are consistent with the idea that NAA reductions in the caudate and ACC may reflect a progression of SCZ, rather than being present in the early stages of the disorder.

On the other hand, we established that CHR had lower tNAA/Cre levels in the rDLPFC relative to HC, with a medium effect size. Although a 3T MRS study reported no difference in NAA in the DLPFC of ultrahigh risk individuals vs. HC [26], our findings are consistent with a meta-analysis showing that high risk individuals had lower NAA/Cre levels (i.e., a 5.7% decrease on average) relative to healthy controls in the PFC, based on which it was concluded that NAA/Cre levels in the PFC could be considered as a biological vulnerability marker of schizophrenia [27].

We also found a significant negative correlation between reduced NAA in the rDLPFC and positive symptom severity in CHR. Specifically, CHR participants who reported worse psychotic symptoms with the SOPS assessment had significantly lower tNAA/Cre in the rDLPFC. These metabolic differences between the CHR group and the HC group could possibly represent putative state biomarkers that reflect the severity of psychosis. In line with this assumption, two recent systematic reviews reported that most studies identified increased NAA concentrations in response to treatment [22,44]. Furthermore, an MRS study investigating the relationship between alterations in metabolite levels in the DLPFC and clinical symptoms of patients with first episode SCZ longitudinally found that a one-year follow-up increase in the NAA/Cr level was detected in the left DLPFC of these patients, which was significantly correlated with the alteration in their positive symptom scores [45]. It remains to be established, though, whether these effects are specific and most prevalent for antipsychotic medications, and if they are associated with an improvement in positive symptoms. Similarly, future longitudinal studies in CHR are needed to confirm whether metabolic deviations, and especially reduced NAA in the DLPFC, are related to the positive psychosis symptoms of these individuals, and whether an increase in DLPFC NAA is associated with the response to antipsychotic treatment.

Additional work is needed to address some of the questions left unanswered by the present study. First, given the exploratory, hypothesis-generating nature of this study, we did not correct the Cho and NAA results for multiple comparisons. Our findings should, therefore, be interpreted with caution, and warrant replication in larger groups of patients. Future studies should also examine these metabolites and their alterations in relation to different stages of SCZ and related psychotic disorders. Second, the results reported here are cross-sectional. Longitudinal studies are, therefore, needed to assess whether lower NAA can predict and track illness trajectory in different stages of psychosis. Third, we found the most NAA alteration in CHR in the right DLPFC. The DLPFC plays a crucial role in regulating cognitive function, particularly working memory. Future studies should, therefore, investigate the relationship between cognition and tNAA/Cre in CHR individuals.

## 4. Materials and Methods

### 4.1. Participants

The study participants comprised twenty-six CHR individuals and thirty-two healthy control subjects.

### 4.2. Recruitment, Eligibility Criteria, and Clinical and Cognition Measurements

This study was approved by the University of Pittsburgh Institutional Review Board. HC and CHR participants were recruited via online and physical advertising efforts within the local community, as well as via referrals from other studies. CHR participants were also recruited from the University of Pittsburgh Medical Center clinical settings, referrals from other clinicians, and outside sources. Written consent from participants over 18 years old, and from parents of participants under 18 years old, was acquired prior to completing study procedures. Participants were informed that their participation was entirely voluntary. Participants were financially compensated for their participation in the study.

Each participant had to meet the following eligibility criteria: (1) be between ages 12 and 35; (2) have no lifetime diagnostic history of neurological disorders or of a head injury resulting in loss of consciousness for longer than a minute; (3) be proficient enough in English to be able to partake in clinical assessments and study procedures; (4) be able to travel to the Western Psychiatric Hospital to participate in study procedures; (5) no pregnancy; (6) have no history within the previous 12 months of alcohol or drug dependence; and (7) be capable of providing written consent. CHR participant eligibility was also assessed on the following criteria: (1) be assessed by the SIPS and meet one of the four prodromal states: (I) genetic risk and functional deterioration state, (II) attenuated positive symptom state, currently progressive, and (III) brief intermittent psychotic state, and (2) have no present diagnosis of a psychotic disorder. HC participants were excluded for the following criteria: (1) having an Axis-1 disorder diagnosis, as assessed by the structured clinical interview for DSM-IV disorders (SCID-IV); (2) having a high risk syndrome diagnosis; and (3) having first-degree relatives with a known diagnosis of a psychotic disorder.

### 4.3. ^1^H MRSI Data Acquisition and Processing

Neuroimaging data were acquired on a 7T Siemens (Siemens Healthcare, Erlangen, Germany) Magnetom scanner using an 8-channel multiple transmit system with a body gradient coil, and an 8 × 2 transceiver array (2 rows, 8 coils/row, Resonance Research Inc., Billerica, MA, USA). A very high order shim (VHOS) insert coil (with a 38 cm inner diameter), providing 3rd and 4th order shims, and partial 5th order shims (Resonance Research Inc.), was used in conjunction with the standard 1st and 2nd order corrections for higher degree/order B0 shimming [46]. The VHOS was used, as it reduces in-plane magnetic field inhomogeneity by ~50% in comparison to what is achieved when using only 1st and 2nd order shims [46]. Planar MRSI data were acquired in a slice angulated along the DLPFC plane using a slice selective J-refocused coherence transfer sequence [47] with TE/TR = 34/1500 ms, matrix size = 24 × 24 over a FOV of 216 mm × 216 mm, and slice thickness = 10 mm (nominal voxel size = 9 mm × 9 mm × 10 mm). Four Hz of Gaussian broadening was used alongside a 100 Hz convolution difference in the spectral domain. A Hanning filter was applied before the Fourier transformation in the spatial domain. This resulted in the in-plane distribution having a full width at half maximum diameter of approximately 16.9 mm. The integrated volume weighted by efficiency is parallel to an unfiltered data set. A frequency-selective inversion recovery preparation module suppressed water and optimized semiselective refocusing pulses [48]. A 1.0 mm isotropic resolution in a FOV of 240 × 240 × 192 mm for anatomic identification was provided by an MP2RAGE sequence [49], with TR of 6000 ms and TI of 80 and 2700 ms. Scout images contained 33 3 mm thick 128 mm × 128 mm slices with in-plane resolution of 1.7 mm. The scout images were matched, and used to weigh and phase the data for coil recombination.

The anatomical brain images (MP2RAGE) were segmented into different cortical and subcortical regions of interest using Freesurfer (FreeSurfer, http://surfer.nmr.mgh.harvard.edu/, accessed on 1 November 2022). The scout image was coregistered to the MP2RAGE using SPM (SPM12, www.fil.ion.ucl.ac.uk/spm, accessed on 1 November 2022). SPM was also used to further transform MP2RAGE into the MNI space to assess the voxel center MNI coordinates and assign voxels to Brodmann areas. For each participant, we selected ROIs from the image that were coregistered into MNI, and confirmed that the placements of these ROIs were in either correct brain regions or Brodmann areas (e.g., for DLPFC, Brodmann area 9 or 46) using MNI152 brain Atlas.

The spectral data were quantified in the range of 1.8 to 4.0 ppm via LCModel [50]. This consisted of default macromolecule components and 14 basis metabolite functions (N-acetylaspartate (NAA), N-acetylaspartylglutamate (NAAG), aspartate, lactate, creatine (Cre), γ-aminobutyric acid (GABA), glucose, glutamate, glutamine, glutathione, glycerophosphorylcholine, phosphorylcholine, myoinositol, and taurine). GAMMA simulations incorporating the effects of the pulse sequence were used to calculate these basic functions [51]. Metabolite ratios (using Cre as the denominator in metabolite ratios) were calculated instead of absolute concentrations to avoid potential quantification errors. These errors could have been introduced by the partial volume effects through the inclusion of cerebrospinal fluid, transmit inhomogeneity due to high field effects, and spatially varying sensitivity due to the use of a receive array. As Cre is a metabolite with a strong signal, reliable chemical shift, and good stability, it is often utilized as an internal reference for other metabolites and for allowing a shorter acquisition time compared to using a water reference. For this study, two neurometabolic ratios were calculated: tNAA/Cre and tCho/Cre. In these ratios, Cre represented the sum of creatine and phosphocreatine, tNAA represented the total of NAA and N-acetylaspartylglutamate (NAAG), and tCho represented the total of major choline-containing compounds, glycerophosphorylcholine and phosphorylcholine.

The LCModel provides Cramer–Rao lower bounds (CRLB) values for each metabolite/neurotransmitter as a representation of the estimate’s uncertainty for any metabolite. More specifically, the CRLB measures data quality by estimating the lowest possible variance for an estimator, and indicating how well the measurement of a given metabolite fits the ideal spectrum for that metabolite. Data with poor spectral quality were filtered out using CRLB values for each metabolite ratio. The exclusion criteria for spectra were CRLB > 10 for the singlet resonances tNAA, Cre, or tCho.

### 4.4. Regions of Interest

**Figure 2:**
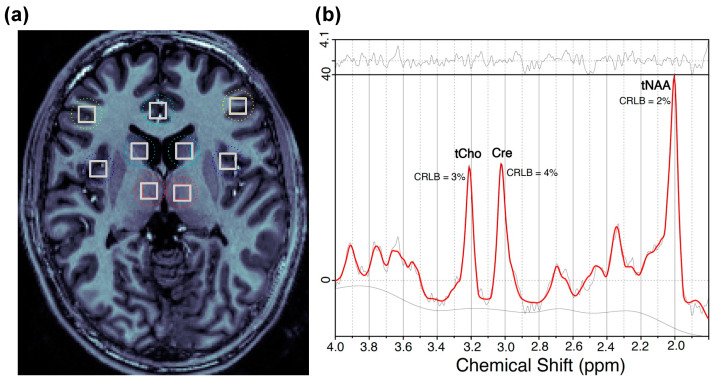
(**a**) Representative location for the regions of interest of a clinical high risk for psychosis (CHR) individual. (**b**) Individual fits for the major compounds of interest; their Cramer–Rao lower bound (CRLB) values of magnetic resonance spectroscopic imaging (MRSI) spectrum are from the right dorsolateral prefrontal cortex (DLPFC) of the CHR individual, the brain of which is shown in part (**a**).

In this study, we investigated tNAA/Cre and tCho/Cre in several cortical and subcortical regions. The cortical regions include the bilateral dorsolateral prefrontal cortex (DLPFC), anterior cingulate cortex (ACC), and bilateral insula. The subcortical ROIs include bilateral caudate and thalamus. Figure 2a represents the locations of selected brain regions. The spectra from those ROIs were then analyzed with LCModel to obtain the final values for each compound. A representative spectrum of a CHR patient is shown in Figure 2b.

### 4.5. Statistical Analysis

A 2-sided Pearson chi-square and an unpaired *t*-test were applied to test age and sex differences between CHR and HC, respectively. An unpaired *t*-test was applied to compare white matter, gray matter, and CSF for each brain region separately between CHR and HC. A one-way analysis of covariance (ANCOVA), with the group as the independent variable, the metabolite level as the dependent variable, and age, sex, gray matter, and white matter as the proportion covariates, was conducted for each metabolite ratio (i.e., tNAA/Cre and tCho/Cre). We calculated Cohen’s effect size for significant findings and post hoc statistical power using the G*Power tool [52].

## 5. Conclusions

In summary, by using 7T MRSI in CHR individuals and HC subjects, in this exploratory study, we found no alterations in the choline level of CHR individuals, while we reported lower NAA in the right DLPFC of CHR, which correlated with their positive symptoms. These findings are in line with previous reports in the early course and chronic patients with SCZ, and suggest that reduced tNAA/Cre in CHR vs. HC may represent a putative molecular biomarker for the risk of psychosis and SCZ that is associated with symptom severity.

## Figures and Tables

**Table 1:** Clinical and demographic measures of the study participants.

Measures	Healthy Control	Clinical High Risk	*p*-Value
Number of Subjects	32	26	-
Sex (# female)	18	15	0.912 ^1^
Average Age ± SD in Years (range)	21.48 ± 4.98 (14–33)	19.93 ± 3.16 (15–27)	0.172 ^2^
SOPS Positive Symptoms (average ± SD)	-	12.19 ± 3.10	-
SOPS Negative Symptoms (average ± SD)	-	11.60 ± 4.34	-
SOPS Disorganized Symptoms (average ± SD)	-	5.00 ± 1.94	-
SOPS General Symptoms (average ± SD)	-	7.68 ± 2.56	-

Abbreviations: SOPS: scale of prodromal symptoms; ^1^ Pearson chi-square 2-sided *p*-value; ^2^ unpaired two-tailed Student’s *t*-test *p*-value.

**Table 2:** Group differences between CHR and HC in tNAA/Cre and tCho/Cre by applying analysis of covariance (ANCOVA) and having the group as the main effect, and age, sex, and gray and white matter fraction as the covariates.

		tCho/Cre		tNAA/Cre	
		F-Stat	*p*-Value	F-Stat	*p*-Value
Right DLPFC	Group	1.086	0.302	4.038	0.049
	Age	0.692	0.410	0.048	0.827
	Sex	0.268	0.607	<0.001	0.999
	GM%	0.058	0.811	1.746	0.192
	WM%	3.330	0.074	2.362	0.131
Left DLPFC	Group	0.436	0.513	2.070	0.158
	Age	2.059	0.156	0.905	0.347
	Sex	0.003	0.954	0.064	0.802
	GM%	0.170	0.683	0.317	0.577
	WM%	2.280	0.139	0.400	0.531
ACC	Group	0.227	0.636	0.253	0.617
	Age	2.557	0.116	0.021	0.887
	Sex	8.289	0.006	0.419	0.520
	GM%	0.144	0.706	0.099	0.755
	WM%	2.265	0.138	<0.001	0.993
Right Caudate	Group	0.723	0.399	1.584	0.214
	Age	0.864	0.357	1.290	0.261
	Sex	6.758	0.012	0.970	0.329
	GM%	0.075	0.785	0.035	0.852
	WM%	0.080	0.778	2.907	0.094
Left Caudate	Group	0.319	0.575	0.115	0.736
	Age	0.280	0.599	0.072	0.790
	Sex	2.121	0.152	0.816	0.371
	GM%	0.523	0.473	0.326	0.571
	WM%	<0.001	0.996	0.702	0.406
Right Insula	Group	0.110	0.742	0.048	0.826
	Age	0.051	0.822	0.095	0.759
	Sex	6.390	0.015	1.703	0.198
	GM%	2.047	0.159	0.317	0.576
	WM%	0.007	0.931	0.428	0.516
Left Insula	Group	0.413	0.523	0.173	0.679
	Age	0.557	0.459	0.184	0.970
	Sex	2.735	0.104	3.316	0.074
	GM%	10.045	0.003	4.232	0.045
	WM%	0.002	0.967	1.401	0.242
Right Thalamus	Group	0.525	0.472	0.351	0.556
	Age	0.558	0.458	0.269	0.606
	Sex	10.316	0.002	0.449	0.506
	GM%	0.166	0.685	0.042	0.839
	WM%	1.960	0.168	0.003	0.953
Left Thalamus	Group	0.523	0.473	0.926	0.341
	Age	0.182	0.671	0.001	0.974
	Sex	6.227	0.016	0.917	0.172
	GM%	2.568	0.115	0.341	0.562
	WM%	2.718	0.106	0.356	0.131

Abbreviations: tCho: glycerophosphorylcholine + phosphorylcholine; tNAA: N-acetylaspartate (NAA) + N-acetylaspartylglutamate (NAAG); DLPFC: dorsolateral prefrontal cortex; ACC: anterior cingulate cortex; GM: gray matter; WM: white matter.

## Data Availability

Data can be requested from the authors.
